# Quantification of *Actaea racemosa* L. (black cohosh) from some of its potential adulterants using qPCR and dPCR methods

**DOI:** 10.1038/s41598-020-80465-0

**Published:** 2021-02-22

**Authors:** Jeevitha Shanmughanandhan, Dhivya Shanmughanandhan, Subramanyam Ragupathy, Thomas A. Henry, Steven G. Newmaster

**Affiliations:** NHP Research Alliance, College of Biological Sciences, University of Guelph, 50 Stone Road East, Guelph, ON N1G 2W1 Canada

**Keywords:** Plant sciences, Plant molecular biology, Biological techniques, PCR-based techniques

## Abstract

The demand for popular natural health products (NHPs) such as Black Cohosh is increasing considerably, which in turn challenges quality assurance (QA) throughout the supply chain. To detect and quantify the target species present in a given NHP, DNA-based molecular techniques such as Real-time quantitative PCR (qPCR) and digital PCR (dPCR) are standard tools in the food and pathogen testing industries. There is a gap in the literature concerning validated quantitative PCR methods for botanicals that can be utilized for QA and good manufacturing practices. The objective of this study is to develop an efficient quantification method using qPCR and dPCR techniques for the detection and quantification of *Actaea racemosa* (Black cohosh) NHPs from its potential adulterants. These developed methods are validated for applicability on commercial NHPs. Species-specific hydrolysis probe assays were designed to analyze the black cohosh NHPs using qPCR and dPCR techniques. The results confirmed that the developed qPCR and dPCR methods are highly precise for identifying and quantifying black cohosh NHPs, indicating their potential applicability in future routine industrial and laboratory testing. This enables a single qPCR test to determine not only the presence of a specific botanical, but also the amount when mixed with an adulterant.

## Introduction

The quality assurance of natural health products relies on quantitative and qualitative analytical techniques. These techniques include analytical chemical methods like HPLC (High-Performance Liquid Chromatography), MS (Mass Spectrometry) and TLC (Thin Layer Chromatography) of which some of these methods can quantitatively detect the presence of chemical entities irrespective of their classification as medicinal plant species or their counterpart contaminants^1^. However, these methods do not indicate the basis of contamination, such as the botanical species identity of the plant material, and, also, the adulterant chemical compounds may not be specified^1,2^. In the past few years, many standardized practices have been followed to ensure proper sourcing of plant materials within the industry based on targeted analytical chemistry that confirms the identity of known standard phytochemicals associated with certain botanical. However, these methods are fit-for-purpose to test for specific chemicals not species concepts and are often inadequate for identifying the target plant species within a complex herbal product^1^. Therefore, more recently, DNA-based molecular biology techniques have been widely used for both qualitative and quantitative detection of biological materials^3,4^. DNA-based analysis techniques are fit-for-purpose to identify species based on phylogenetic concepts and genetic inheritance principles founded upon evolutionary biology. These molecular diagnostic techniques are appropriate to verify species ingredients because DNA is more species-specific and less subjective to environmental/manufacturing conditions such as high temperature that change the chemical profile of a NHP^5^. Previous research has demonstrated that manufacturing process may reduce the quantity of DNA and break DNA into smaller fragments, but it is often still detected^6,7^. PCR reactions can be performed successfully with minimal amounts of DNA, and this facilitates high specificity and sensitivity of target species assays^8^ on small fragment of DNA in low quantity.

In recent years, there has been a significant increase in the sale and usage of natural health products, which presents a challenge for sourcing authentic botanical ingredients from the supply chain. One of the more commonly adulterated botanicals is black cohosh (*Actaea racemosa)*. *Actaea racemosa* products are prone to a high level of mislabeling /contamination with other species such as *A. pachypoda, A. rubra,* and *A. cimicifuga*. Various qualitative analyses and DNA-based molecular studies have been proposed to identify the presence of adulterants in *A. racemosa* NHPs^9–17^. Currently, there are no studies that have focused on quantifying *A. racemosa* and its adulterants using qPCR and digital droplet PCR techniques. Our research centers on quantifying the amount of *A. racemosa* present in each NHP sample.

Methods such as qPCR and dPCR have been gaining popularity to quantify the amount of target and unknown adulterants^18^ in food and feed samples^19^, meat products^20,21^, fish products^22^, microbial ecology^23^, GMOs^24^. This is the first study to quantify botanicals using qPCR and dPCR methods. Both of these techniques are more advanced and sensitive than the conventional PCR technique. The qPCR technique slightly varies from conventional PCR; in qPCR, amplicons are detected in real-time during each cycle with the help of fluorescent signals. The fluorescence signal produced during the exponential period will be directly proportional to the DNA concentration^25^. Double-stranded DNA-binding fluorescent dyes and probes used in the qPCR experiments produce fluorescent signals when DNA amplification occurs. Unlike fluorescent dyes, fluorescent probes bind only to the defined target DNA, which provides specificity^25^. The qPCR technique can be used to perform two types of quantification: (1) absolute quantification and (2) relative quantification. Absolute quantification involves generating a standard calibration curve, where the unknown sample is quantified based on the known sample quantity. However, the accuracy of the standards is critical in using this method for quantification. Relative quantification involves normalization of the target gene with an endogenous or an exogenous calibrator^26^. Therefore, known standards are not necessary. One of the critical phases in relative quantification analysis is data normalization. It is performed to minimize the sample-to-sample variations like when using different matrices and run-to-run differences^27–29^. The normalization of the target gene for an endogenous or an exogenous calibrator is performed by using mathematical models based on the comparison of cycle threshold (Ct) values^26^. The exogenous calibrator can be spiked into the sample and allows absolute and accurate quantification of the amplicons even with a low concentration of the target DNA (< 1 ng/reaction setup)^26,30–32^. The exogenous calibrator can be advantageous in measuring the PCR bias, as the quantity of template spiked into the sample is known. Some concerns with the use of using exogenous calibrators are that specific unknown samples may contain inhibitors that potentially reduce the efficiency of the PCR reaction, or that formation of primer dimers may hinder the specific amplicon formation^33^. Endogenous controls, on the other hand, are assumed to remain constant between cells of different tissues and under different environmental conditions^34^. The appropriate type of control (either exogenous or endogenous) will vary depending on the environmental conditions^35–40^.

Digital droplet PCR is the third generation of PCR techniques; it is highly sensitive and more accurate than qPCR. It is the amalgamation of PCR along with fluorescence-based detection. This technique is used for absolute quantification of target DNA through excessive dilution and partitioning of the sample into numerous droplets (~ 20,000 droplets) that are then subjected to end-point PCR^34,41^. After the PCR, separation of these droplet partitions into positive (target DNA present in the droplets) and negative (target DNA absent in the droplets) corresponds to the initial number of the target DNA. The droplet generation, based on the oil–water emulsion, allows easy partitioning of the samples into numerous droplets^42^. The generated positive and negative droplets are analysed using Poisson distribution, in which determination of the average number of targets is calculated by the ratio of the positive droplets to the total droplets generated^42^.

Although various PCR methods have been validated for species identity of botanical ingredients, there is gap in the literature for validated quantitative PCR methods for botanicals that can be utilized for QA and good manufacturing practices. The objective of this study is to compare and validate two different techniques, quantitative PCR (qPCR) and digital droplet PCR, and to quantify the *Actaea racemosa* target species in commercial NHPs. The accuracy and applicability of the methods were verified using experimental mixtures of black cohosh samples and its potential adulterants and from commercially available products, respectively.

## Results

### DNA extraction efficiency, quality and quantity

The DNA extracted from 1 g of the target, non-targets and, external calibrator samples used for engineered DNA mixtures yielded between 20 and 78 ng/μl DNA. All the sample mixtures were found to have yielded DNA concentrations between 40 and 84 ng/μl; subsequently, each sample was normalized to 1 ng/μl for further analyses.

### Analytical specificity, repeatability, limits of detection and quantification

**Figure 1:**
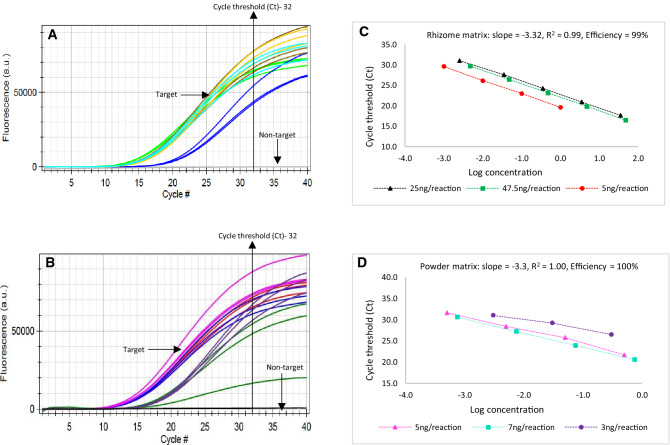
In (**A**, **B**), the X-axis represents the threshold cycle, and Y-axis represents the fluorescent units. Evaluating the specificity of *A. racemosa* qualitative PCR assay using 20 target samples and 20 non-target samples using bCUBE instrument. All target samples successfully amplified with Ct values ranging from 18 to 22, for different sample matrices [Standard Botanical Reference Material (SBRM), rhizome and powder]. Non-target samples did not amplify. (**C**, **D**) Efficiency of *A. racemosa* qualitative PCR assay for rhizome and powder matrices. (**C**) Six 10-fold dilution series were prepared from 25 ng, 47.5 ng and 5 ng DNA as starting concentration. Each dilution was tested in triplicate. The amplification efficiency was 99% with R^2^ = 0.99. (**D**) Six 10-fold dilution series were prepared from 5 ng, 7 ng and 3 ng DNA as starting concentration. Each dilution was tested in triplicate. The amplification efficiency was 100% with R^2^ = 1.00.

The *A. racemosa* assay, after optimization for analytical specificity, analytical sensitivity, repeatability and reproducibility using qPCR and dPCR instruments, showed 100% specificity (Fig. 1) and 99% efficiency. Analytical sensitivity for the qPCR assay was found to be 0.25 × 10^–2^ ng^43^. The exogenous calibrator sample tested for the same parameters in both the instruments displayed 99.3% specificity, and analytical sensitivity was 0.25 × 10^–2^ ng for the qPCR assay.

Duplicate dPCR reactions were carried out for testing the specificity of both the *A. racemosa* and exogenous calibrator assays, and it was found that both the assays were specific. The lowest limit of quantification of the *A. racemosa* assay was found to be 8 copies/μl. The lowest limit of detection for the *A. racemosa* assay and the exogenous calibrator assay was found to be 2.4 copies/μl and 2 copies/μl, respectively. According to botanical pharmacopeia monographs and industry standards, up to 2–5%, foreign material can be present in a sample, and it would not be considered to be contaminated^44^. Additionally, due to various undetermined inaccuracies such as sampling error, DNA quality and quantity, limits the accurate quantification of herbal mixtures that are present in amounts lower than 10%. Hence the lowest limit of quantification of adulterants/contaminants for this study was set to 10%^45^.

### qPCR

#### Quantification of *A. racemosa* target DNA from the DNA mixtures

Using Pfaffl method^26^, the measured proportions of *A. racemosa* in the DNA mixtures (100%, 90%, 80%, 50%, 10%) using LightCycler 480 (LC480) platform were 100%, 97%, 85%, 49%, and 10%, respectively (see Supplementary Table S1).

Similarly, by using the bCUBE portable qPCR instrument, the proportions obtained were 100%, 87%, 81%, 48% and 9%, respectively (see Supplementary Table S2). A standard deviation (std. dev) of ≤ 0.1 between the Ct values yielded an approximate target percentage using bCUBE (see Supplementary Table S2).

#### Quantification of *A. racemosa* target DNA from the sample mixtures

The *A. racemosa* sample mixtures were quantified using two different qPCR platforms. The ratio of *A. racemosa* in the sample mixtures (100%, 90%, 80%, 50%, 10%) measured using LC-480 and their relative standard deviation (RSD) were 100% (RSD = 0.51%), 97% (RSD = 0.14%) , 76% (RSD = 0.17%), 54% (RSD = 0.58%), and 11% (RSD = 1.25%), respectively (see Supplementary Table S3). The standard deviation (Std. dev) for the triplicate analysis of mixtures of both the *A. racemosa* assay and the exogenous calibrator assay ranged from 0.03 to 0.25 using LC-480 (see Supplementary Table S3). The relative standard deviation percent was calculated to determine the precision of the assay. The average RSD% calculated for the sample mixtures (100%, 90%, 80%, 50%, 10%) by performing six parallel quantifications using the *A. racemosa* assay were 0.20%, 0.45%, 0.36%, 0.27%, and 1.47%, respectively. RSDs of 0.54%, 0.76%, 0.43%, 0.33%, and 1.08% were obtained for the same samples when the exogenous calibrator assay was performed. The individual and the average relative standard deviation (RSD) were < 2% for all the mixtures, which shows that the precision of measurement using LC-480 was within the technical range^46^. From the results, it is evident that using the Pfaffl method, the quantification results obtained are more precise for the sample mixtures using the LC-480 instrument.

When the same samples were tested with the bCUBE instrument, the ratios were obtained as 100% (RSD = 0.20%), 68% (RSD = 2.42%), 79% (RSD = 0.24%), 53% (RSD = 0.47%), and 13% (RSD = 1.13%), for the sample mixtures 100%, 90%, 80%, 50%, and 10%, respectively (see Supplementary Table S4). The standard deviation for the mixtures with the *A. racemosa* assay and the exogenous calibrator assay using the bCUBE instrument ranged from 0.05 to 0.48 (see Supplementary Table S4). Similar to the LC-480 tests, the relative standard deviation percent was calculated for six parallel quantifications using the bCUBE instrument and the average RSDs for 100%, 90%, 80%, 50%, and 10% sample mixtures were 0.48%, 0.64%, 0.16%, 0.19%, 0.50% using the *A. racemosa* assay and 0.68%, 0.85%, 0.43%, 0.58%, 0.98% using the exogenous calibrator assay respectively. The individual and the average RSDs were ≤ 1%.

#### Quantification of commercial products

The percentages of commercial raw material and powdered samples were determined using the raw material (root powder) as a positive calibrator. From Supplementary Table S5, using LC-480, the measured values for samples CS1, CS2 (labelled as pure *A. racemosa*) were 100% and 93.3%, respectively. Whereas using bCUBE, it was observed to be 98% and 92.2%, respectively (see Supplementary Table S6). From the results obtained, the proposed quantification method is highly applicable to commercial samples.

The measured value for the sample CS4 was 33% (see Supplementary Table S5) and 32% (see Supplementary Table S6) using the LC-480 and bCUBE platforms, respectively. The deviation of the sample CS4 from the original ratio of 28% was observed to be 18% and 11% using LC480 and bCUBE platforms, respectively.

### dPCR

#### Optimization of dPCR assay

A temperature gradient was performed using 8 temperatures (55.0 °C, 55.8 °C, 57.3 °C, 59.4 °C, 61.8 °C, 63.9 °C, 65.3 °C, and 66.0 °C) on the thermal cycler to determine the optimum annealing temperature for the dPCR assay with different dilution factors such as 10, 20, 30, 40 for both *A. racemosa* and the exogenous calibrator assays. The amount of template DNA used in the dPCR reaction mixture was optimized by diluting 1 ng of template DNA with dilution factors of 40 and 10 for the *A. racemosa* assay and the exogenous calibrator assay, respectively. Target template concentrations ranging from 0.0125 to 0.5 ng/μl and temperatures between 61 °C and 66 °C yielded the highest fluorescent distinction between positive and negative droplets. Hence, an annealing temperature of 64 °C was set as the ideal temperature for the *A. racemosa* assay (see Supplementary Fig. S1). Similarly, an annealing temperature of 58 °C was determined for the exogenous calibrator assay, using a temperature gradient between 55 °C and 60 °C with template DNA concentrations ranging between 0.125 ng/μl and 0.5 ng/μl.

#### Restriction digestion of gDNA

The efficiency and the precise quantification of the target DNA copy number were notably improved by the addition of a restriction enzyme to the reaction mixture^47^. For the *A. racemosa* assay, the addition of HaeIII (2units/μl) restriction enzyme resulted in the clear separation of positive and negative droplets and reduced the number of rainy droplets (see Supplementary Fig. S2).

#### Quantification of *A. racemosa* target DNA from DNA mixtures

Table 1 shows the ratio of copy numbers found for the engineered DNA mixtures using the *A. racemosa* and exogenous calibrator assays; the results obtained were 100%, 93%, 77%, 51% and 10% with respect to the originally engineered DNA ratios of 100%, 90%, 80%, 50% and 10%. The RSD % was within the acceptable range (≤ 25%) for all the ratios. The calculated % bias did not show any odd deviations.

**Table 1:** Evaluation of the ratio of *A. racemosa* present in the engineered DNA mixture using dPCR.

*A. racemosa* probe				Exogenous calibrator probe			*N*	*K*	Percent % obtained
Sample	Copies/μl	Avg. copies/μl	Std. dev	Copies/μl	Avg. copies/μl	Std. dev			
*A. racemosa*100%	313	310	3.83	156	162	7.70	1.00	310	100
	308			167					
90%	313	314	1.41	171	177	7.42	1.09	288	93
	315			182					
80%	272	278	7.81	188	187	1.07	1.16	240	77
	283			186					
50%	170	172	2.21	167	177	13.66	1.09	158	51
	174			186					
10%	37	36	1.87	181	181	0.65	1.12	32	10
	34			182					

Standard deviations (Std. dev) for the *A. racemosa* assay and the exogenous calibrator assay are shown. Columns *N* represents normalised value and *K* represents normalised copy numbers of the samples.

#### Quantification of *A. racemosa* target DNA from the sample mixtures

A similar procedure for the evaluation of the *A. racemosa* target copy numbers from the different engineered sample mixtures was followed (see Supplementary Table S7). The measured values of *A. racemosa* for different mixture ratios (100%, 90%, 80%, 50% and 10%) were found to be 100% (RSD = 0.6%), 88% (RSD = 1.4%), 74% (RSD = 1.3%), 35% (RSD = 0.00%) and 11% (RSD = 4.8%), respectively. The average RSD obtained by performing six parallel quantifications were 0.67%, 0.88%, 0.44%, 1.31% and 3.30% respectively for the (100%, 90%, 80%, 50% and 10%) sample mixtures. Among these observed values, the underestimation of the positive droplets led to the deviation of the ratio for the 50% sample mixture, with an estimated value of 35%.

## Discussion

The preliminary step in all the quantification experiments is to determine the DNA extraction efficiency, quality (see Supplementary Table S8) and quantity. DNA quantification is essential to normalize target DNA used for the quantification experiments^48^, which was performed using the Qubit 3.0 Fluorometer (Invitrogen, Carlsbad, CA). For our study, the *ITS* region was found to be a suitable region to discriminate *A. racemosa* from the non-targets^49^. *ITS* is a region of the nuclear genome present in multiple copies, and it displays concerted evolution^50^. Due to these multiple copies, precise quantification of *A. racemosa* was challenging. qPCR and dPCR are both susceptible to reaction inhibitors such as secondary structure formation in primers and reagents, which will completely inhibit the reaction. Therefore, to nullify these undetected problems, an exogenous calibrator sample was used for data normalization^33,48,51,52^. The exogenous calibrator selected for this study was obtained from chloroplast genome where, the target amplicon sequence is located between 34,215 and 34,313 bp of the GenBank accession: MN561034.1. The developed assays were optimized for its analytical specificity, reliability, limits of detection and quantification. All the dPCR reactions were carried out in duplicates, and the lowest limit of quantification of adulterants/contaminants for this study was set to 10%^45^. Also, according to botanical pharmacopeia monographs and industry standards, up to 2–5%, foreign material can be present in a sample, and it would not be considered to be contaminated^44^. Additionally, due to various undetermined inaccuracies such as sampling error, DNA quality and quantity, limits the accurate quantification of herbal mixtures that are present in amounts lower than 10%.

Absolute quantification methods quantify the target DNA in comparison to a standard curve, rather than the total quantity of the target genes present within the sample^53^. Relative quantification does not need such standard curves, and hence, it was selected as a suitable method for quantifying the actual amount of target species (*A. racemosa*) from the mixtures for all the qPCR quantifications. During qPCR technique of sample mix quantification, Initially*,* a total of 1.5 g of *A. racemosa*, non-target and exogenous calibrator samples were mixed in different ratios: 100% (1 g of *A. racemosa* + 500 mg exogenous calibrator), 90% (900 mg *A. racemosa* + 100 mg non-target + 500 mg exogenous calibrator), 50% (500 mg of each *A. racemosa*, non-target and exogenous calibrator) and 10% (100 mg *A. racemosa* + 900 mg non-target and 500 mg exogenous calibrator), respectively, for DNA extraction and quantification. The quantification ratio of *A. racemosa* measured for the mixtures 90%, 50% and 10% were 76%, 71% and 32%, respectively. When these sample mixtures were quantified using the LC- 480 instrument, the percentage of target detection was not accurate, and this inaccuracy may be attributed to sampling error. The DNA yield and quality can be greatly reduced because of the presence of inhibitors in the samples when the sample mixes are prepared from a smaller amount of tissue. Since the results were not accurate with the LC-480 instrument, further quantification was not performed using the bCUBE instrument. Further experiments were carried out after increasing the tissue amount to 15 g in total (see “Materials and methods”) to reduce the sampling error bias and improve the accuracy of quantification. Thus, the quality of the quantification data obtained was significantly enhanced, and this provided improved insight into the quantification results (see Supplementary Table S8). After the successful quantification of engineered mixtures of various percentages (100%, 90%, 80%, 50%, 10%) of *A. racemosa* along with non-targets and an exogenous calibrator, the practical application of the method was evaluated on commercial samples. Unlike the engineered mixtures, which mainly consisted of raw materials like rhizome and seeds, the finished products were highly processed. To check the feasibility of this method on different types of matrices (such as raw materials, powders, powdered extracts) 4 commercial samples (CS1, CS2, CS3, CS4) of different matrices were analyzed using the developed qPCR method using both the LC-480 (see Supplementary Table S5) and bCUBE (see Supplementary Table S6) platforms. 100% target *A. racemosa* raw material sample with a spiked-in exogenous calibrator was used as the control sample to determine the actual percentages of commercial samples. Using both the LC-480 and bCUBE instruments, calculation of the percentages for CS3, CS4 samples (Ct values =  ~ 33, 34 and 35, 36 respectively) using 100% target raw material (Ct value =  ~ 17 and 18) was inappropriate as normalization was not possible for samples with significantly varying Ct values. Hence, the extract sample normalization was done by taking 100% *A. racemosa* extract and spiked-in exogenous calibrator as the control. Hence 100% CS3 was taken as the control and normalized against sample CS4, which yielded a better result. Hence it can be established that similar sample matrices of the control and test samples are essential considerations for accurate quantification. Finally, these results confirm the applicability and feasibility of the developed qPCR assays using the LC-480 and bCUBE platforms.

Quantification of *A. racemosa* was performed using the formulae (1, 2, 3, 4) mentioned in the qPCR and dPCR data analysis section (see supplementary data analysis). dPCR discriminates even minor changes in concentration with increased precision and reliability. We were able to obtain approximate results since the engineered DNA mixtures were prepared after the DNA extraction. This will significantly reduce the PCR inhibitors. With improved sensitivity, tolerance to PCR inhibitors, and less susceptibility to differences in PCR efficiencies, dPCR can be used for accurate detection and quantification of target species. When sample mix quantification was performed, among the observed values, the underestimation of the positive droplets led to the deviation of the ratio for the 50% sample mixture, with an estimated value of 35%. Sometimes the underestimation of target DNA concentration can be caused by a decrease in PCR efficiency, which results in the reduction of fluorescent signals of positive droplets. Increased amounts of PCR inhibitors can also contribute to poor assay efficiency and lower fluorescent signals, which makes it more difficult to differentiate between positive and negative droplets^46^.

The summary of the results obtained from the sample mixtures analyzed using qPCR and dPCR methods with a minimum of three repetitions is shown in Table 2. The real-time quantitative PCR (qPCR) method uses a linear relationship between the threshold cycle value and initial target DNA copy numbers present in the qPCR reaction. Digital PCR (dPCR) measures the number of positive droplets produced, which is assumed to be proportional to the amount of *A. racemosa* present in the mixture. In this study, the relative quantification of *A. racemosa* present in the mixtures was performed using an exogenous calibrator. The efficiencies of both *A. racemosa* and exogenous calibrator assays were 100.0% and 99.3%, respectively.

**Table 2:** Limit of quantification for engineered sample mixtures of *A. racemosa* using qPCR and dPCR.

Actual mix ratio of *A. racemosa* (%)	qPCR						dPCR		
	LC-480			bCUBE					
	Measured ratio (%)	RSD (%)	Bias (%)	Measured ratio (%)	RSD (%)	Bias (%)	Measured ratio (%)	RSD (%)	Bias (%)
100%	100	0.20	0.00	100	0.48	0.00	100	0.67	0.00
90%	98	0.45	8.90	93	0.64	3.52	88	0.88	− 2.20
80%	74	0.36	− 7.90	77	0.16	− 3.75	74	0.44	− 11.70
50%	54	0.27	8.00	53	0.19	6.33	36	1.30	− 28.00
10%	11	1.47	10.00	12	0.50	15.00	11	3.30	10.00

According to the results, both the qPCR and dPCR methods showed relative standard deviations (RSDs) ≤ 5% for sample mix quantification and showed good accuracy. Compared to the bias of > 5% in LC-480, the bCUBE qPCR platform showed bias < 5% for the mixtures 80% and 90%, which shows that bCUBE out-performs LC-480 in terms of accurate estimation of the quantity of *A. racemosa*. In comparison, dPCR shows an underestimation of the quantity with a negative bias of − 11% and − 2% for 80% and 90%, respectively. However, for the 50% *A. racemosa* mixture, the bias % for dPCR was higher than 25%, while that of qPCR (LC-480 and bCUBE) was less than 10%*.* For the 10% *A. racemosa* mixture, the bias was ≥ 10% for both qPCR and dPCR (Table 2). As dPCR is more sensitive than qPCR, inhibitors present in either of the samples used to produce the mix might have reduced the efficiency of the assay, making it challenging to differentiate the positive and negative droplets. The heterogeneous nature of the samples likely contributed to the occurrence of unidentifiable errors that might affect the qPCR and dPCR assay efficiencies, such as sampling error, the presence of complex contaminants, or the quality of the DNA (level of fragmentation). Hence, by improving some initial sample processing steps like the DNA extraction method as well as the appropriate selection of calibrators, these types of errors were minimized (see Supplementary Table S8).

According to our results, the method of precise quantification of the mixtures was enhanced by reducing the unidentifiable errors, and hence we conclude that the qPCR method of quantification performed using the bCUBE platform has lower bias and higher precision when compared to the LC-480 platform. While comparing dPCR over qPCR, both the techniques show similar results for quantifying plant-based NHP mixtures, when provided with quality DNA.

## Conclusion

Several DNA based molecular diagnostic tools are being developed for the qualitative and quantitative assessment of natural health products. In recent years, NGS methods are gaining popularity, as they are employed in the authentication of natural health products^54^. However, there are challenges like low sequence coverage, repetitive sequences, DNA amplification bias caused during PCR amplification affecting the target species identification^44^. Raw botanicals come from farms and are harvested, shipped and stored in the same distribution and storage centers in which small oligo fragments of DNA called incidental DNA fragments (contaminants and adulterants) of many plant species may mix with main ingredient species. In such cases, PCR bias that occurs during NGS experiments may result in over-estimation of the incidental DNA than the targeted DNA. This finally leads to potentially misleading and inconsequential taxon identification that has no relevance to the NHP as it is in such small amounts. Hence, it is difficult to develop standard operating protocols (SOPs) for identifying NHPs using NGS methods. On the other hand, SOPs have been developed and are successfully employed in identifying target species using qPCR and dPCR methods. Genetic markers such as microsatellites are also being used in various evolutionary biology studies for their high mutation rates. Yet, it is difficult to develop these markers as it requires construction of genomic libraries with enriched repeated motifs for most of the species^55^. Also, it is expensive and time consuming. There are several other potential drawbacks such as the presence of stutter bands, null alleles heterologous amplicons and lack of universality for target species identification. Whereas barcode markers used in this study generates high quality DNA barcodes which improves target species identification^3,55^.

In this study, we have demonstrated the identification and quantification of natural health products (NHPs) using qPCR and dPCR methods. Also, the developed quantification assay can be specified as a standard to facilitate industry (from supply chain to end-product manufacturing) use in routine laboratory sample identification and quantification testing. Real-time qPCR has widely been used in the quantification of nucleic acids in various species. However, the idea of quantification of NHPs using a portable qPCR platform (bCUBE) and a highly sensitive and reliable dPCR platform are investigated here for the first time. While dPCR is highly sensitive, accurate and reliable, qPCR is more cost-effective. The strategy tested in this study involved the use of an exogenous calibrator sample in both qPCR and dPCR quantification, to normalize the target copy numbers. Engineered DNA and sample mixtures, along with commercial samples, were used to test and prove the applicability of this assay. The formulae used in this study for calculating the target DNA percentage are convenient and understandable for the quantification of *A. racemosa* and other NHPs.

In conclusion, these quantification methods could also be used for regular NHP testing, since NHPs are prone to frequent contamination and are consumed worldwide. The major limitation of this developed assay is it must be further optimized for detecting the target species present in extracts because the presence of a very low amount of amplifiable DNA will result in Ct value > 32. To further improve the performance and the reliability of the assay, an extensive market survey of the commercial samples will be performed using both qPCR and dPCR methods.

## Materials and methods

### Sample preparation

**Figure 2:**
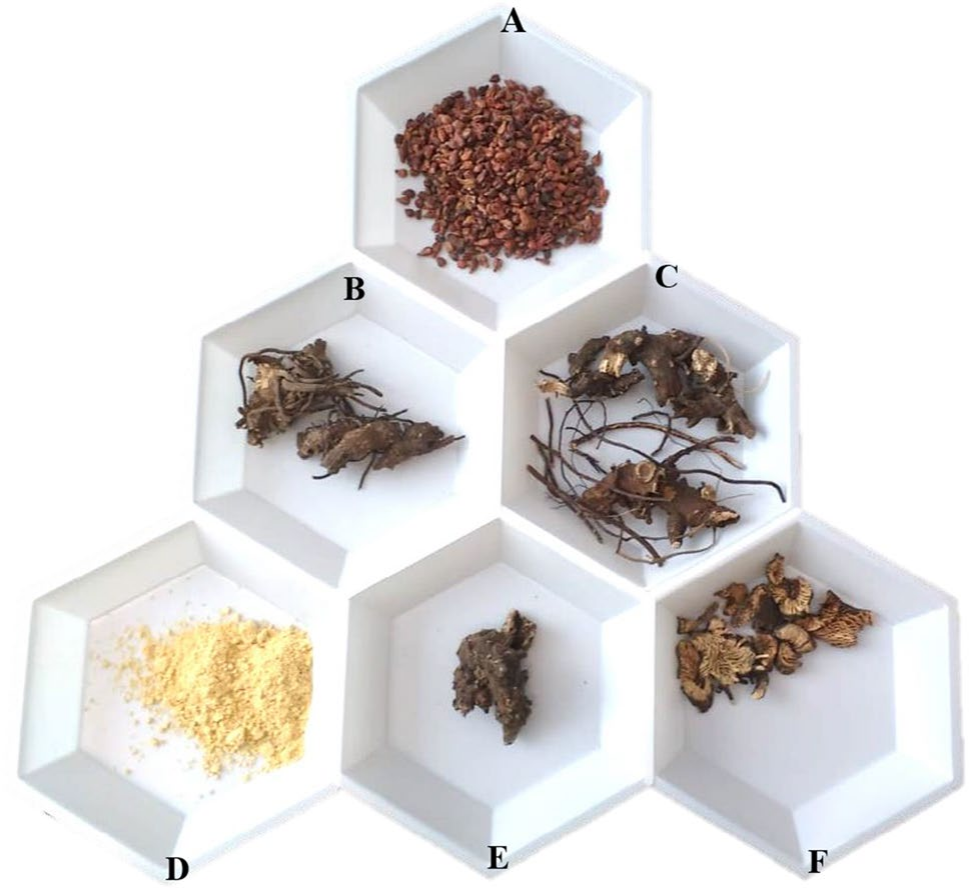
Samples used for preparing engineered sample and DNA mixtures. (**A**) Raw material of the exogenous calibrator used in this study. (**B**,**C**) *A. racemosa* raw material used for preparing various mixture ratios. (**D**–**F**) Nontargets/adulterants used for preparing engineered sample mixtures.

Samples selected were based on their availability and according to the industrial manufacturing process (Fig. 2). For simulating adulteration, different proportions of the target, non-target/adulterants and exogenous/external calibrator were mixed in two methods: (1) DNA mix and (2) Sample mix.

### DNA mix

1 ng of DNA extracted from 1 g of each sample: target (*Actaea racemosa)*, non-targets (*Hypericum perforatum, Zingiber officinale*, *A. pachypoda, A. cimicifuga*) and exogenous calibrator sample were used to prepare the DNA mix in the ratio as mentioned in Table 3.

**Table 3:** Engineered DNA mix ratios prepared for the quantification of target *A. racemosa.*

Sample name		Target % (μl)	Non-target % (μl)	Exogenous calibrator amount (μl)	Total (μl)
EC + T (100%)	VR-3	50	–	4	54
EC + T (90%) + NT (10%)	VRZ-1	45	5	4	54
EC + T (80%) + NT (20%)	VRZ-2	40	10	4	54
EC + T (50%) + NT (50%)	VRZ-3	25	25	4	54
EC + T (10%) + NT (90%)	VRZ-4	5	45	4	54

*DNA T* target DNA, *NT* non-target DNA, *EC* exogenous calibrator DNA.

### Sample mix

Raw materials such as root/rhizome/seed were used to prepare the engineered sample mixture. Actual samples of *A. racemosa* and non-target/adulterants were mixed at the ratios of 90% (9 g + 1 g), 80% (8 g + 2 g), 50% (5 g + 5 g), 10% (1 g + 9 g) respectively (see Supplementary Table S9). An exogenous calibrator sample of 5 g was spiked in uniformly to each of the sample mixtures. Finally, raw material tissues were grounded using the instrument Magic Bullet Blender and DNA extraction was carried out using a total of 15 g of each sample mix. In addition to these sample mixtures, four commercial samples containing *A. racemosa* were tested to determine the applicability of the method used for quantifying engineered mixtures. The retail sample matrices ranged from raw material to powdered extracts. The samples contained 100% *A. racemosa* and combinations of different plant species (see Supplementary Table S10).

### DNA extraction and quantitation

The DNA extraction was performed using the Nucleospin Plant II Midi kit (Macherey–Nagel GmbH & Co. KG, Düren, Germany) with minor modification in the initial step of cell lysis. Instead of the PL1/PL2 cell lysis buffers provided with the kit, manually prepared buffers consisting of 2% CTAB (Cetyl trimethylammonium bromide) (see Supplementary Table S11) and 2% PVP (Polyvinylpyrrolidone) (2 g in 100 mL distilled water) were used. 500 μl of 2% CTAB and 200 μl of 2% PVP were used for 1 g of sample, and if this was not sufficient for the samples to dissolve, CTAB and PVP were increased proportionally. All remaining steps were followed according to the manufacturer’s instructions to obtain high-quality DNA. DNA quantification of all the samples was performed using the Qubit 3.0 Fluorometer (Invitrogen, Carlsbad, CA).

### Primers and probes design

*Insilico* analysis of barcode markers such as *ITS, matK, rbcL, psbA* were performed to design species-specific probe assay for *A. racemosa.* Among these barcode markers, the *ITS* region distinguished *A. racemosa* from its commonly used adulterants and closely related phylogenetic species*. A. racemosa* assay primers and probe was designed according to general guidelines and recommendations^56,57^ using Integrated DNA technologies’ PrimerQuest tool (see Supplementary Table S13). The reference sequences used in the design of primers and probe were from a vouchered library with herbarium samples of known provenance archived at the NHP Research Alliance OAC Herbarium, University of Guelph and NCBI database (see Supplementary Table S12).

Exogenous calibrator assay primers and probe were designed following the alignment of chloroplast gene sequences^49^ of the exogenous calibrator and closely related phylogenetic species according to general guidelines and recommendations^56,57^. The reference sequences used in the design of primers and probe were from a vouchered library with herbarium samples of known provenance archived at the NHP Research Alliance OAC Herbarium, University of Guelph and NCBI database.

### Assay optimization for quantification

qPCR assays for the target *A. racemosa* and exogenous calibrator were carried out using LC-480 and bCUBE instruments. For quantification studies, extensive calibration and optimization of the exogenous calibrator were performed, including testing for its analytical sensitivity, analytical specificity, repeatability and reproducibility on both qPCR instruments. According to the recommendations for validation of real-time PCR methods for molecular diagnostic identification of botanicals^58^, all the experiments were performed in triplicate.

According to the digital MIQE guidelines^48^, dPCR optimization for *A. racemosa* and the exogenous calibrator assays were performed to determine their analytical specificity, analytical sensitivity (limit of quantification = LOQ and limit of detection = LOD), repeatability and reproducibility. All the experiments performed contained technical duplicates^48^. LOQ was determined by evaluating the lowest limit of quantification using the engineered *A. racemosa* DNA mixtures in ratios ranging from 90 to 10%. LOD was established by detecting the lowest amount of detectable DNA of the pure target sample by preparing two-fold dilution series of the *A. racemosa* sample with concentration ranging from 0.025 to 0.00078 ng/μl.

### qPCR

qPCR reactions were performed on a LightCycler 480 instrument (LC480) (Roche Diagnostics, Indianapolis, IN) and with a portable Hyris bCUBE 2.0 instrument (Hyris Global Diagnostics Ltd, Milan, Italy) using the SensiFAST Probe No-ROX Kit (Bioline, London, United Kingdom). PCR reactions were carried out in a final volume of 20 μl, including 5 μl of template DNA, 10 μl of SensiFAST Probe No-ROX mix, 2 μl of 10X primer and probe and brought upto 20 μl with nuclease-free water. Real-time PCR cycling conditions for both LC480 bCUBE instruments consisted of initial denaturation time for 3 min at 95 °C followed by 35–40 amplification cycles with denaturation for 30 s at 95 °C and an annealing/extension for 30 s at 66 °C.

Following the same procedure for the exogenous calibrator assay, PCR reactions were performed in a final volume of 20 μl, including 5 μl of template DNA, 10 μl of SensiFAST Probe No-ROX mix, 1 μl of 5X primer and probe and brought up to 20 μl with nuclease-free water. Real-time PCR cycling conditions for both LC480 and bCUBE instruments consisted of initial denaturation time for 2 min at 95 °C followed by 35–40 amplification cycles with denaturation for 10 s at 95 °C and an annealing/extension for 20 s at 58 °C.

### dPCR

dPCR reactions were performed in a final volume of 22 μl, including 5 μl of template, 11 μl of 2X ddPCR Supermix for Probes (No dUTP), 1.1 μl of 10X primer and probe, 1 μl of HaeIII restriction enzyme (2 units/μl) and brought up to 22 μl with nuclease-free water. The PCR reaction mixture was then mixed with the Automated Droplet Generation oil for Probes (Bio-Rad) in a DG32 Cartridge (Bio-Rad) and loaded into the QX200 AutoDG Droplet Generator (Bio-Rad) to generate PCR droplets. From each droplet mix, 22 μl was transferred to a dPCR 96 Well plate (Bio-Rad). A pierceable foil heat-seal was used to seal the plate using PX PCR Plate Sealer (Bio-Rad). Real-time PCR cycling conditions consisted of initial denaturation time for 10 min at 95 °C followed by 44 amplification cycles with denaturation for 30 s at 94 °C and an annealing/extension for 1 min at 64 °C, followed by elongation at 72 °C for 30 s and a final incubation at 98 °C for 10 min and holding at 10 °C until the reading time. The results from the generated droplets were analyzed using the QX200 Droplet Reader, and each droplet’s fluorescent signal was evaluated using its associated Quanta-Soft Analysis pro (1.0.596) software (Bio-Rad). The results recorded are as copies/μl with confidence intervals of 95%. The droplet count generated from each well containing at least 10,000 or more were analyzed further.

dPCR reactions for the exogenous calibrator were performed in a final volume of 22 μl, including 5 μl of templates, 11 μl of 2X ddPCR Supermix for Probes (No dUTP), 1.1 μl of 10X primer and probe, and brought up to 22 μl with nuclease-free water. Restriction digestion of the external/exogenous calibrator gene was not performed**.** Real-time PCR cycling conditions consisted of initial denaturation time for 10 min at 95 °C followed by 44 amplification cycles with denaturation for 30 s at 94 °C and an annealing/extension for 1 min at 58 °C, followed by elongation at 72 °C for 30 s and a final incubation at 98 °C for 10 min and holding at 10 °C until the reading time.

### dPCR thermal gradient and dilution factor optimization

Optimal annealing temperature and the dilution factor were analyzed for both *A. racemosa* and the exogenous calibrator to perform the dPCR assays. To choose the optimal annealing temperature, sixteen dPCR reactions containing the same amount of DNA were performed, and the annealing temperature was varied in a gradient ranging from 55 to 66 °C for 1 min extension time. Different dilution factors ranging from 10 to 60 were used in sixteen dPCR reactions to determine the appropriate dilution factor. All the reactions were carried out using Gradient C1000 Touch Thermal Cycler (Bio-Rad, Mississauga, ON, Canada) to optimize the *A. racemosa* assay and exogenous calibrator assay.

### Enzymatic restriction digestion of gDNA

Enzymatic restriction digestion of *A. racemosa* gDNA was carried out with HaeIII (New England Biolabs, USA) to obtain good quantity and quality of the DNA for dPCR reactions. Restriction digestion was used to separate the gene copies, ensuring proper random partitioning into droplets. It was also used to reduce sample viscosity and to increase template accessibility improving assay performance. The non-specific amplifications are greatly reduced which can be seen in the form of rainy droplets (see Supplementary Fig. S2). To obtain a final concentration of the enzyme as two units/μl, 40 μl of 1X CutSmart buffer prepared from 10X stock solution and 10 μl of the enzyme HaeIII (10,000 units/ml) were mixed, and 1 μl of the diluted enzyme was added to digest 1 ng/μl of DNA present in the dPCR reaction mixture.

### Data analysis

Data analysis for quantifying the target species was performed based on the efficiency and cycle threshold for qPCR, and dPCR quantification was performed based on the normalization factor (see Supplementary Data analysis).

## Supplementary Information

Supplementary Information
