# Supplementary material for: Quantification of Actaea racemosa L. (black cohosh) from some of its potential adulterants using qPCR and dPCR methods
Source: Sci Rep. 2021 Feb 22;11:4331. doi: 10.1038/s41598-020-80465-0 (PMC7900226; doi:10.1038/s41598-020-80465-0)
Supplement: Supplementary file 1 — Supplementary Information [file 41598_2020_80465_MOESM1_ESM.docx]

**Quantification of *Actaea racemosa* L. (black cohosh) from some of its potential adulterants using qPCR and dPCR methods.**

Jeevitha Shanmughanandhan, Dhivya Shanmughanandhan*, Subramanyam Ragupathy, Thomas A Henry and Steven G Newmaster.

NHP Research Alliance, College of Biological Sciences, University of Guelph, Guelph, Ontario, Canada, N1G 2W1

Corresponding author e-mail: [shanmugd@uoguelph.ca](mailto:shanmugd@uoguelph.ca)

**Supplementary Information**

**List of Tables**

[**Table S1:** Evaluating the engineered DNA mix percentages using the qPCR method on the LC-480 instrument. The mean Ct and the standard deviation (std.dev) were calculated from triplicate reactions by performing the *A. racemosa* and exogenous calibrator assays. The ratio of specific *A. racemosa* DNA to total DNA was derived directly from the formula of Pfaffl. 2](#_Toc55892543)

[**Table S2:** Evaluating the engineered DNA mix percentages using the qPCR method on the bCUBE instrument. The mean Ct and the standard deviation (std.dev) were calculated from triplicate reactions by performing the *A. racemosa* and exogenous calibrator assays. The ratio of specific DNA to total DNA was derived directly from the formula of Pfaffl. 3](#_Toc55892544)

[**Table S3:** Evaluating the engineered sample mix percentages using the qPCR method on the LC-480 instrument. The mean Ct and the standard deviation (std.dev) were calculated from triplicate reactions by performing the *A. racemosa* and external control assays. The ratio of specific *A. racemosa* DNA to total DNA was derived directly from the formula of Pfaffl. 4](#_Toc55892545)

[**Table S4:** Evaluating the engineered sample mix percentages using the qPCR method on the bCUBE instrument. The mean Ct and the standard deviation (std.dev) were calculated from triplicate reactions by performing the *A. racemosa* and external control assays. The ratio of specific *A. racemosa* DNA to total DNA was derived directly from the formula of Pfaffl. 5](#_Toc55892546)

[**Table S5:** Evaluating the established quantification strategy on commercial samples using the LC-480 instrument. 100% *A. racemosa* with spiked-in exogenous calibrator as the control sample was used for calculating the percentages 6](#_Toc55892547)

[**Table S6:** Evaluating the established quantification strategy on commercial samples using the bCUBE instrument. 100% *A. racemosa* with spiked-in exogenous calibrator as the control sample was used for calculating the percentages. 7](#_Toc55892548)

[**Table S7:** Evaluation of the ratio of *A. racemosa* present in the engineered sample mixture using dPCR. Standard deviation (Std. dev) for the *A. racemosa* assay and the exogenous calibrator assay are shown. 8](#_Toc55892549)

[**Table S8:** The measured ratio by LC-480 of *A. racemosa* present when two different amounts of the sample mixtures are used. 8](#_Toc55892550)

[**Table S9:** Engineered sample mix ratios prepared for the quantification of target *A. racemosa* DNA. T- target DNA, NT- non-target DNA, EC- exogenous calibrator DNA. Target, non-target, and exogenous calibrator sample tissues were mixed in different ratios, and then DNA extraction was performed. 9](#_Toc55892551)

[**Table S10:** List of commercial samples tested. 9](#_Toc55892552)

[**Table S11**: Chemical composition of 2% CTAB cell lysis buffer (50ml). 10](#_Toc55892553)

[**Table S12**: Target and Non-target species used in this study. Amplifiable amount of DNA was obtained from all the samples listed 10](#_Toc55892554)

[**Table S13:** Sequences of primers and probe used in the development of *A. racemosa* assay. 14](#_Toc55892555)

**List of Figures**

[**Figure S1:** Fluorescence amplitude plotted against the annealing temperature gradient. All the positive droplets with PCR amplification are in blue dots while the negative droplets are grey coloured. The vertical yellow line shows the number of wells in which the temperature gradient was setup. The reactions were performed with the following gradient temperatures: 55°C, 55.8°C, 57.3 °C, 59.4 °C, 61.8 °C, 63.9 °C, 65.3 °C, and 66.0°C and dilution factor of 40. Appropriate annealing temperature ranges between 61.8°C and 66°C. 14](#_Toc39664637)

[**Figure S2: a)** Optimization of *A. racemosa* assay at various temperatures and dilutions without the addition of restriction enzyme shows no differentiation between positive (blue coloured dots) and negative droplets (grey coloured dots). **b)** Optimization of *A. racemosa* assay at various temperatures and dilutions with the addition of restriction enzyme HaeIII shows clear separation between positive (blue coloured dots) and negative droplets (grey coloured dots). 15](#_Toc39664638)

[**Data Analysis 1: qPCR and dPCR quantification** 16](#_Toc39665039)

**List of symbols and abbreviations**

NHPs Natural health products

PCR Polymerase chain reaction

qPCR quantitative Real-time PCR

dPCR Digital PCR

gDNA Genomic DNA

SBRM Standard biological reference material

NHPRA Natural health product research alliance

LOD Limit od detection

LOQ Limit of quantification

Ct Cycle threshold

LC-480 LightCycler®480

E Amplification efficiency

R^2^ Coefficient of determination

pg Picogram

ng Nanogram

mg Milligram

nM Nanomolar

g Gram

µl Microlitre

Std. dev/SD Standard deviation

*p* Positive droplets

PVP Polyvinylpyrrolidone

CTAB Cetyl trimethylammonium bromide

RSD Relative standard deviation

**Tables**

**Table S1:** Evaluating the engineered DNA mix percentages using the qPCR method on the LC-480 instrument. The mean Ct and the standard deviation (std.dev) were calculated from triplicate reactions by performing the *A. racemosa* and exogenous calibrator assays. The ratio of specific *A. racemosa* DNA to total DNA was derived directly from the formula of Pfaffl.

| ***A. racemosa* probe** | | | | **Exogenous calibrator probe** | | | **Normalized** | **Percent (%)**  **obtained** |
| --- | --- | --- | --- | --- | --- | --- | --- | --- |
| **Samples** | **CT**  **value** | **Avg. CT** | **Std.dev** | **CT**  **value** | **Avg. CT** | **Std.dev** |  |  |
| *A. racemosa*  100% | 18.58 | 18.79 | 0.21 | 22.86 | 22.80 | 0.10 | 1 | 100 |
|  | 18.8 |  |  | 22.68 |  |  |  |  |
|  | 19 |  |  | 22.86 |  |  |  |  |
| 90% | 19.06 | 18.92 | 0.13 | 22.84 | 22.89 | 0.08 | 0.97 | 97 |
|  | 18.81 |  |  | 22.98 |  |  |  |  |
|  | 18.89 |  |  | 22.84 |  |  |  |  |
| 80% | 19.08 | 19.03 | 0.11 | 22.84 | 22.80 | 0.03 | 0.85 | 85 |
|  | 18.9 |  |  | 22.78 |  |  |  |  |
|  | 19.1 |  |  | 22.78 |  |  |  |  |
| 50% | 19.99 | 19.83 | 0.22 | 22.76 | 22.80 | 0.04 | 0.49 | 49 |
|  | 19.58 |  |  | 22.83 |  |  |  |  |
|  | 19.91 |  |  | 22.8 |  |  |  |  |
| 10% | 22.72 | 22.19 | 0.46 | 22.85 | 22.84 | 0.15 | 0.1 | 10 |
|  | 21.86 |  |  | 22.99 |  |  |  |  |
|  | 22 |  |  | 22.69 |  |  |  |  |

**Table S2:** Evaluating the engineered DNA mix percentages using the qPCR method on the bCUBE instrument. The mean Ct and the standard deviation (std.dev) were calculated from triplicate reactions by performing the *A. racemosa* and exogenous calibrator assays. The ratio of specific DNA to total DNA was derived directly from the formula of Pfaffl.

| ***A. racemosa* probe** | | | | **Exogenous calibrator probe** | | | **Normalized** | **Percent (%)**  **Obtained** |
| --- | --- | --- | --- | --- | --- | --- | --- | --- |
| **Samples** | **CT**  **value** | **Avg. CT** | **Std.dev** | **CT**  **value** | **Avg. CT** | **Std.dev** |  |  |
| *A. racemosa*  100% | 19.09 | 19.14 | 0.05 | 23.04 | 22.95 | 0.15 | 1 | 100 |
|  | 19.18 |  |  | 22.77 |  |  |  |  |
|  | 19.16 |  |  | 23.03 |  |  |  |  |
| 90% | 19.23 | 19.19 | 0.06 | 22.89 | 22.80 | 0.09 | 0.87 | 87 |
|  | 19.12 |  |  | 22.78 |  |  |  |  |
|  | 19.22 |  |  | 22.72 |  |  |  |  |
| 80% | 19.44 | 19.39 | 0.06 | 22.92 | 22.90 | 0.11 | 0.81 | 81 |
|  | 19.39 |  |  | 23 |  |  |  |  |
|  | 19.33 |  |  | 22.79 |  |  |  |  |
| 50% | 20.12 | 20.12 | 0.05 | 22.93 | 22.86 | 0.09 | 0.48 | 48 |
|  | 20.08 |  |  | 22.84 |  |  |  |  |
|  | 20.17 |  |  | 22.76 |  |  |  |  |
| 10% | 22.56 | 22.50 | 0.05 | 22.82 | 22.82 | 0.02 | 0.09 | 9 |
|  | 22.46 |  |  | 22.83 |  |  |  |  |
|  | 22.48 |  |  | 22.8 |  |  |  |  |

**Table S3:** Evaluating the engineered sample mix percentages using the qPCR method on the LC-480 instrument. The mean Ct and the standard deviation (std.dev) were calculated from triplicate reactions by performing the *A. racemosa* and external control assays. The ratio of specific *A. racemosa* DNA to total DNA was derived directly from the formula of Pfaffl.

| ***Actaea racemosa* probe** | | | | **Exogenous calibrator probe** | | | **Normalized** | **Percent (%)**  **obtained** | **RSD %** |
| --- | --- | --- | --- | --- | --- | --- | --- | --- | --- |
| **Samples** | **CT**  **value** | **Avg. CT** | **Std.dev** | **CT**  **value** | **Avg. CT** | **Std.dev** |  |  |  |
| *A. racemosa*  100% | 17.58 | 17.67 | 0.09 | 20.2 | 20.21 | 0.06 | 1 | 100 | 0.51 |
|  | 17.68 |  |  | 20.16 |  |  |  |  |  |
|  | 17.76 |  |  | 20.27 |  |  |  |  |  |
| 90% | 18.04 | 18.04 | 0.03 | 20.55 | 20.54 | 0.03 | 0.97 | 97 | 0.14 |
|  | 18.07 |  |  | 20.57 |  |  |  |  |  |
|  | 18.02 |  |  | 20.51 |  |  |  |  |  |
| 80% | 17.85 | 17.82 | 0.03 | 19.89 | 19.96 | 0.11 | 0.76 | 76 | 0.17 |
|  | 17.82 |  |  | 19.9 |  |  |  |  |  |
|  | 17.79 |  |  | 20.09 |  |  |  |  |  |
| 50% | 19.37 | 19.33 | 0.11 | 21 | 20.98 | 0.09 | 0.54 | 54 | 0.58 |
|  | 19.41 |  |  | 20.89 |  |  |  |  |  |
|  | 19.2 |  |  | 21.06 |  |  |  |  |  |
| 10% | 20.27 | 20.30 | 0.25 | 19.69 | 19.76 | 0.14 | 0.11 | 11 | 1.25 |
|  | 20.57 |  |  | 19.92 |  |  |  |  |  |
|  | 20.07 |  |  | 19.68 |  |  |  |  |  |

**Table S4:** Evaluating the engineered sample mix percentages using the qPCR method on the bCUBE instrument. The mean Ct and the standard deviation (std.dev) were calculated from triplicate reactions by performing the *A. racemosa* and external control assays. The ratio of specific *A. racemosa* DNA to total DNA was derived directly from the formula of Pfaffl.

| ***Actaea racemosa* probe** | | | | **Exogenous calibrator probe** | | | **Normalized** | **Percent (%)**  **obtained** | **RSD %** |
| --- | --- | --- | --- | --- | --- | --- | --- | --- | --- |
| **Samples** | **CT**  **value** | **Avg. CT** | **Std.dev** | **CT**  **value** | **Avg. CT** | **Std. dev** |  |  |  |
| *A. racemosa*  100% | 19.19 | 19.15 | 0.04 | 20.87 | 20.99 | 0.16 | 1 | 100 | 0.20 |
|  | 19.12 |  |  | 21.17 |  |  |  |  |  |
|  | 19.13 |  |  | 20.93 |  |  |  |  |  |
| 90% | 20.51 | 19.95 | 0.48 | 21.27 | 21.23 | 0.11 | 0.68 | 68 | 2.42 |
|  | 19.65 |  |  | 21.1 |  |  |  |  |  |
|  | 19.7 |  |  | 21.31 |  |  |  |  |  |
| 80% | 19.33 | 19.28 | 0.05 | 20.92 | 20.77 | 0.15 | 0.79 | 79 | 0.24 |
|  | 19.27 |  |  | 20.75 |  |  |  |  |  |
|  | 19.24 |  |  | 20.63 |  |  |  |  |  |
| 50% | 20.61 | 20.59 | 0.10 | 21.56 | 21.52 | 0.05 | 0.53 | 53 | 0.47 |
|  | 20.49 |  |  | 21.52 |  |  |  |  |  |
|  | 20.68 |  |  | 21.47 |  |  |  |  |  |
| 10% | 21.78 | 21.96 | 0.25 | 21.1 | 20.89 | 0.18 | 0.13 | 13 | 1.13 |
|  | 22.13 |  |  | 20.77 |  |  |  |  |  |
|  |  |  |  | 20.81 |  |  |  |  |  |

.

**Table S5:** Evaluating the established quantification strategy on commercial samples using the LC-480 instrument. 100% *A. racemosa* with spiked-in exogenous calibrator as the control sample was used for calculating the percentages

| **Samples** | **A*ctaea racemosa***  **probe** | | | **Exogenous calibrator probe** | | | **Normalized** | **Percent (%)**  **obtained** |
| --- | --- | --- | --- | --- | --- | --- | --- | --- |
|  | **CT**  **value** | **Avg. CT** | **Std. dev** | **CT value** | **Avg. CT** | **Std. dev** |  |  |
| *A. racemosa*  100% | 17.76 | 17.76 | 0.0 | 20.32 | 20.32 | 0.00 | 1.00 | 100 |
|  | 17.76 |  |  | 20.32 |  |  |  |  |
| CS1 (100%) | 17.66 | 17.66 | 0.01 | 20.37 | 20.38 | 0.014 | 1.00 | 100 |
|  | 17.65 |  |  | 20.39 |  |  |  |  |
| CS2(100%) | 17.73 | 17.74 | 0.01 | 21.88 | 21.87 | 0.014 | 1.08 | 93.3 |
|  | 17.74 |  |  | 21.86 |  |  |  |  |
| CS3(100%) | 33.54 | 33.59 | 0.06 | 18.01 | 18.04 | 0.042 | 1.00 | 100 |
|  | 33.63 |  |  | 18.07 |  |  |  |  |
| CS4(28%) | 35.92 | 35.90 | 0.04 | 18.73 | 18.74 | 0.007 | 0.33 | 33 |
|  | 35.87 |  |  | 18.74 |  |  |  |  |

**Table S6:** Evaluating the established quantification strategy on commercial samples using the bCUBE instrument. 100% *A. racemosa* with spiked-in exogenous calibrator as the control sample was used for calculating the percentages.

| **Samples** | ***Actaea racemosa* probe** | | | **Exogenous calibrator probe** | | | **Normalized** | **Percent (%)**  **obtained** |
| --- | --- | --- | --- | --- | --- | --- | --- | --- |
|  | **CT**  **value** | **Avg.**  **CT** | **Std.**  **dev** | **CT**  **value** | **Avg.**  **CT** | **Std.**  **dev** |  |  |
| *A. racemosa*  100% | 18.75 | 18.72 | 0.05 | 20.97 | 20.97 | 0.01 | 1.00 | 100 |
|  | 18.68 |  |  | 20.96 |  |  |  |  |
| CS1(100%) | 18.62 | 18.65 | 0.04 | 21.33 | 21.33 | 0.01 | 1.02 | 98 |
|  | 18.68 |  |  | 21.32 |  |  |  |  |
| CS2(100%) | 18.66 | 18.65 | 0.01 | 22.64 | 22.66 | 0.02 | 1.08 | 92.2 |
|  | 18.64 |  |  | 22.67 |  |  |  |  |
| CS3(100%) | 34.01 | 34.09 | 0.11 | 18.86 | 18.90 | 0.06 | 1.00 | 100 |
|  | 34.17 |  |  | 18.94 |  |  |  |  |
| CS4(28%) | 36.36 | 36.39 | 0.04 | 19.55 | 19.55 | 0.01 | 0.32 | 32 |
|  | 36.42 |  |  | 19.54 |  |  |  |  |

**Table S7:** Evaluation of the ratio of *A. racemosa* present in the engineered sample mixture using dPCR. Standard deviation (Std. dev) for the *A. racemosa* assay and the exogenous calibrator assay are shown.

| ***A. racemosa* probe** | | | | **Exogenous calibrator probe** | | | ***N*** | ***K*** | **Percent (%)**  **obtained** | **RSD (%)** |
| --- | --- | --- | --- | --- | --- | --- | --- | --- | --- | --- |
| **Sample** | **Copies/μl** | **Avg.**  **copies/μl** | **Std. dev** | **Copies/μl** | **Avg.**  **copies/μl** | **Std. dev** |  |  |  |  |
| *A.*  *racemosa*  100% | 336 | 335 | 2.12 | 568 | 569 | 0.71 | 1.0 | 334.50 | **100** | **0.6** |
|  | 333 |  |  | 569 |  |  |  |  |  |  |
| 90% | 317 | 314 | 4.24 | 600 | 605 | 7.07 | 1.06 | 295.06 | **88** | **1.4** |
|  | 311 |  |  | 610 |  |  |  |  |  |  |
| 80% | 327 | 324 | 4.24 | 742 | 743 | 1.41 | 1.31 | 247.91 | **74** | **1.3** |
|  | 321 |  |  | 744 |  |  |  |  |  |  |
| 50% | 140 | 140 | 0.00 | 682 | 685 | 4.24 | 1.20 | 116.19 | **35** | **0.00** |
|  | 140 |  |  | 688 |  |  |  |  |  |  |
| 10% | 100 | 104 | 4.95 | 1646 | 1666 | 27.58 | 2.93 | 35.33 | **11** | **4.8** |
|  | 107 |  |  | 1685 |  |  |  |  |  |  |

**Table S8:** The measured ratio by LC-480 of *A. racemosa* present when two different amounts of the sample mixtures are used.

| **Actual Samples** | **The measured ratio of *A. racemosa* (%)** | | | |
| --- | --- | --- | --- | --- |
|  | **1.5g Sample quantity** | **Bias (%)** | **15g Sample quantity** | **Bias (%)** |
| *A. racemosa* 90% | 76 | -15.55 | 97 | 7.77 |
| 50% | 71 | 42 | 54 | 8 |
| 10% | 32 | 220 | 11 | 10 |

**Table S9:** Engineered sample mix ratios prepared for the quantification of target A. racemosa DNA. T- target DNA, NT- non-target DNA, EC- exogenous calibrator DNA. Target, non-target, and exogenous calibrator sample tissues were mixed in different ratios, and then DNA extraction was performed.

| **Sample name** | | **Target % (g)** | **Non-target % (g)** | **Exogenous**  **calibrator amount (g)** | **Total (g)** |
| --- | --- | --- | --- | --- | --- |
| EC+T (100%) | VR-3 | 10 | - | 5 | 15 |
| EC+T (90%) + NT (10%) | VRZ-1 | 9 | 1 | 5 | 15 |
| EC+ T (80%) + NT (20%) | VRZ-2 | 5 | 5 | 5 | 15 |
| EC+ T (50%) + NT (50%) | VRZ-3 | 8 | 2 | 5 | 15 |
| EC + T (10%) + NT (90%) | VRZ-4 | 1 | 9 | 5 | 15 |

**Table S10:** List of commercial samples tested.

| **Sample name** | **Matrix** | **Amount of *A. racemosa* present (%)** | **Other species mentioned on the label** |
| --- | --- | --- | --- |
| CS1 | Raw material | 100 | NA |
| CS2 | Powder | 100 | NA |
| CS3 | Powdered extract | 100 | NA |
| CS4 | Powdered extract | 28 | *Angelica sinensis, Vitex agnus castus, Oryza sativa, Citrus sinensis* |

**^NA^ Not applicable**

**Table S11**: Chemical composition of 2% CTAB cell lysis buffer (50ml).

| **Chemical components** | **Amount (g)** |
| --- | --- |
| 2% CTAB | 1 |
| 20mM EDTA | 0.37 |
| 1.4M NaCl | 4.09 |
| 100mM Tris HCl | 0.789 |

**Table S12**: Target and Non-target species used in this study. Amplifiable amount of DNA was obtained from all the samples listed

| **Botanical Name** | **Family** | **Sample code** | **Type of sample** | **Type of material** | **Source** | **Reference sequences Id** |
| --- | --- | --- | --- | --- | --- | --- |
| *A. racemosa* | Ranunculaceae | AR-01 | Target | SBRM | NHPRA-OAC  herbarium | NHPRA database/ AR-01 |
| *A. racemosa* | Ranunculaceae | AR-02 | Target | Rhizome |  | NHPRA database/ AR-02 |
| *A. racemosa* | Ranunculaceae | AR-03 | Target | Rhizome |  | NHPRA database/ AR-03 |
| *A. racemosa* | Ranunculaceae | AR-04 | Target | Rhizome |  | NHPRA database/ AR-04 |
| *A. racemosa* | Ranunculaceae | AR-05 | Target | Rhizome |  | NHPRA database/ AR-05 |
| *A. racemosa* | Ranunculaceae | AR-06 | Target | Rhizome |  | NHPRA database/ AR-06 |
| *A. racemosa* | Ranunculaceae | AR-07 | Target | Rhizome |  | NHPRA database/ AR-07 |
| *A. racemosa* | Ranunculaceae | AR-08 | Target | Powder |  | NHPRA database/ AR-08 |

| **Botanical Name** | **Family** | **Sample code** | **Type of sample** | **Type of material** | **Source** | **Reference sequences Id** |
| --- | --- | --- | --- | --- | --- | --- |
| *A. racemosa* | Ranunculaceae | AR-09 | Target | Powder | various industrial manufacturers | NHPRA database/ AR-09 |
| *A. racemosa* | Ranunculaceae | AR-10 | Target | Powder |  | NHPRA database/ AR-10 |
| *A. racemosa* | Ranunculaceae | AR-11 | Target | Powder |  | NHPRA database/ AR-11 |
| *A. racemosa* | Ranunculaceae | AR-12 | Target | Powder |  | NHPRA database/ AR-12 |
| *A. racemosa* | Ranunculaceae | AR-13 | Target | Powder |  | NHPRA database/ AR-13 |
| *A. racemosa* | Ranunculaceae | AR-14 | Target | Powder |  | NHPRA database/ AR-14 |
| *A. racemosa* | Ranunculaceae | AR-15 | Target | Powdered Extract |  | NHPRA database/ AR-15 |
| *A. racemosa* | Ranunculaceae | AR-16 | Target | Powdered Extract |  | NHPRA database/ AR-16 |
| *A. racemosa* | Ranunculaceae | AR-17 | Target | Powdered Extract |  | NHPRA database/ AR-17 |
| *A. racemosa* | Ranunculaceae | AR-18 | Target | Powdered Extract |  | NHPRA database/ AR-18 |
| *A. racemosa* | Ranunculaceae | AR-19 | Target | Powdered Extract |  | NHPRA database/ AR-19 |
| *A. racemosa* | Ranunculaceae | AR-20 | Target | Powdered Extract |  | NHPRA database/ AR-20 |

| **Botanical Name** | **Family** | **Sample code** | **Type of sample** | **Type of material** | **Source** | **Reference sequences Id** |
| --- | --- | --- | --- | --- | --- | --- |
| *A. pachypoda* | Ranunculaceae | NT-01 | Non-Target | Rhizome | NHPRA-OAC  herbarium | NHPRA database/ NT-01 |
| *A. cimicifuga* | Ranunculaceae | NT-02 | Non-Target | Rhizome |  | NHPRA database/ NT-02 |
| *A. rubra* | Ranunculaceae | NT-03 | Non-Target | Leaf |  | NHPRA database/ NT-03 |
| *Panax ginseng* | Araliaceae | NT-04 | Non-Target | Powder | various industrial manufacturers | GenBank/KM207674.1 |
| *Gingko biloba* | Gingkoaceae | NT-05 | Non-Target | Powder |  | GenBank/EF372233.1 |
| *Curcuma longa* | Zingiberaceae | NT-06 | Non-Target | Powder |  | GenBank/JQ409956.1 |
| *Valeriana officinalis* | Caprifoliaceae | NT-07 | Non-Target | Powder |  | GenBank/EU796889.1 |
| *Hypericum perforatum* | Hypericaceae | NT-08 | Non-Target | Powder |  | GenBank/FJ694215.1 |
| *Zingiber officinale* | Zingiberaceae | NT-09 | Non-Target | Powder |  | GenBank/KR816715.1 |
| *Hydrastis canadensis* | Ranunculaceae | NT-10 | Non-Target | Powder |  | GenBank/MF349151.1 |
| *Arthrospira platensis* | Phormidiaceae | NT-11 | Non-Target | Powder |  | GenBank/AY724775.1 |
| *Equisetum sp.* | Equisetaceae | NT-12 | Non-Target | Powder |  | GenBank/AF448794.1 |

| **Botanical Name** | **Family** | **Sample code** | **Type of sample** | **Type of material** | **Source** | **Reference sequences Id** |
| --- | --- | --- | --- | --- | --- | --- |
| *Olea europaea* | Oleaceae | NT-13 | Non-Target | Powder |  | GenBank/KF805102.1 |
| *Foeniculum vulgare* | Apiaceae | NT-14 | Non-Target | Powder |  | GenBank/AY581806.1 |
| *Brassica oleracea var. capitata* | Brassicaceae | NT-15 | Non-Target | Powder |  | GenBank/GQ891867.1 |
| *Eleutherococcus senticosus* | Araliaceae | NT-16 | Non-Target | Powder |  | GenBank/AF077885.1 |
| *Cinnamomum verum* | Lauraceae | NT-17 | Non-Target | Powder |  | GenBank/KX766399.1 |
| *Astragalus membranaceus* | Fabaceae | NT-18 | Non-Target | Powder |  | GenBank/EF685968.1 |
| *Caulophyllum thalictroides* | Berberidaceae | NT-19 | Non-Target | Powder |  | GenBank/L77159.1 |
| *Camellia sinensis* | Theaceae | NT-20 | Non-Target | Powder |  | GenBank/FJ004886.1 |

**Table S13:** Sequences of primers and probe used in the development of A. racemosa assay.

| **Name** | **Sequence** | **Size, bp** | **Annealing temperature (Ta),**  **°C** |
| --- | --- | --- | --- |
| Actra-118- ITSF | CCGAGTCCTCTTTTGGGCAC | 20 | 58 |
| Actra-118- ITSR | ACGACAAGGCTACGCGTCTT | 20 | 59 |
| Actra- Probe1 | AATATTGGTCCTCGACGACAATCGTCGC | 28 | 61 |

**Figures**

**Figure S1:** Fluorescence amplitude plotted against the annealing temperature gradient. All the positive droplets with PCR amplification are in blue dots while the negative droplets are grey coloured. The vertical yellow line shows the number of wells in which the temperature gradient was setup. The reactions were performed with the following gradient temperatures: 55°C, 55.8°C, 57.3 °C, 59.4 °C, 61.8 °C, 63.9 °C, 65.3 °C, and 66.0°C and dilution factor of 40. Appropriate annealing temperature ranges between 61.8°C and 66°C.


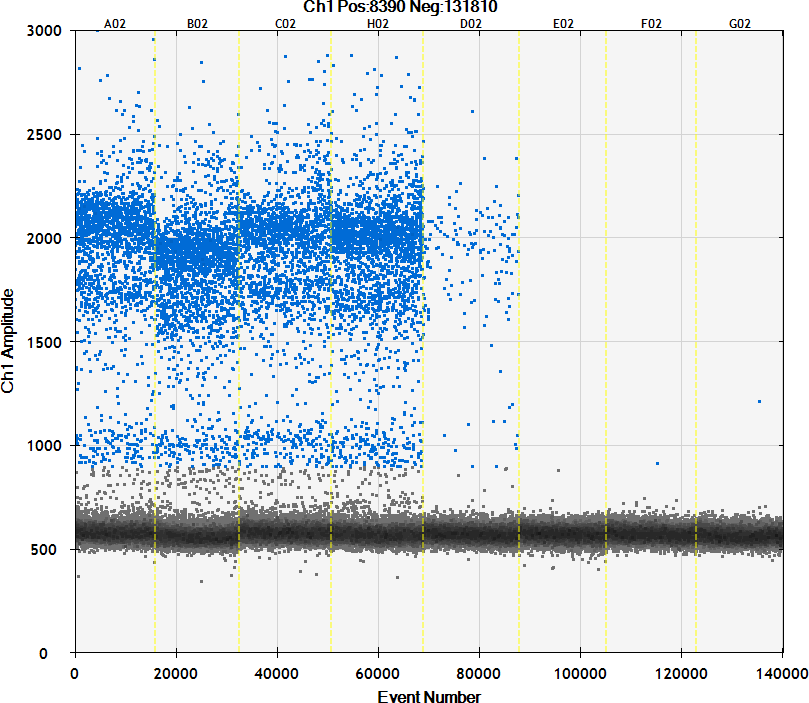


**66**°C

**65.3**°C

**59.4**°C

**57.3**°C

**55**°C

**55.8**°C

**61.8**°C

**63.9**°C

**Figure S2: a)** Optimization of *A. racemosa* assay at various temperatures and dilutions without the addition of restriction enzyme shows no differentiation between positive (blue coloured dots) and negative droplets (grey coloured dots). **b)** Optimization of *A. racemosa* assay at various temperatures and dilutions with the addition of restriction enzyme HaeIII shows clear separation between positive (blue coloured dots) and negative droplets (grey coloured dots).


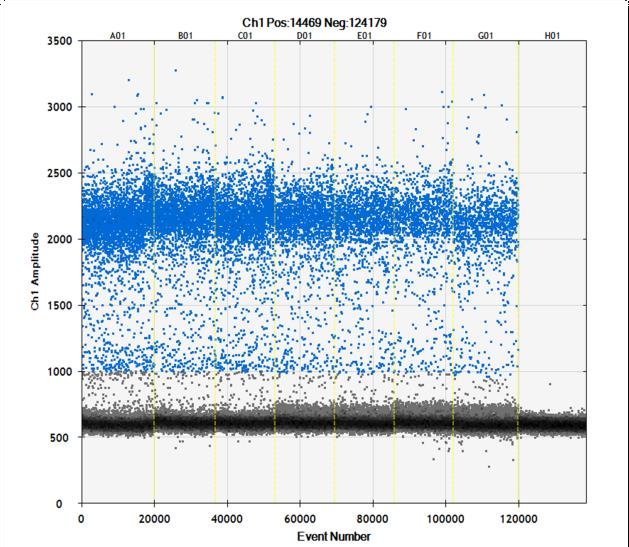

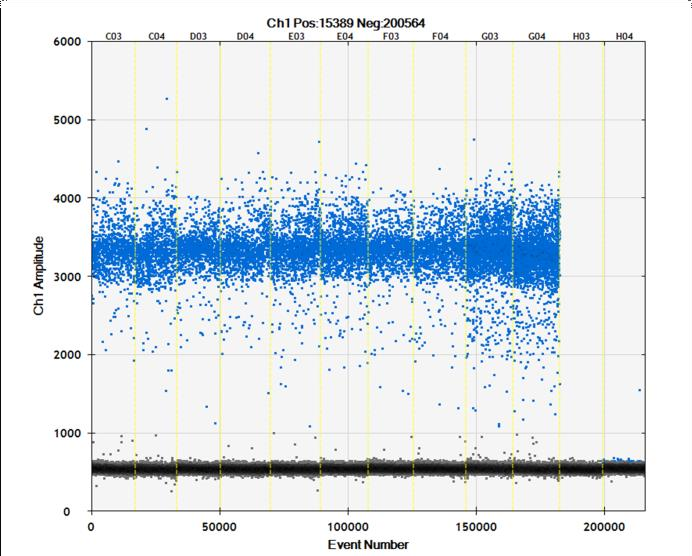


2a

2b

**Data Analysis 1: qPCR and dPCR quantification**

**qPCR quantification based on the efficiency and cycle threshold (Ct).** The qPCR relative quantification analyses were performed according to MIQE guidelines for the validation of real-time PCR methods^1,2^ and using Pfaffl formula^3^.

**dPCR quantification based on normalization factor.** Droplet fluorescence data were analyzed with Quanta-Soft Analysis pro (1.0.596) software (Bio-Rad) using confidence intervals of 95% and recorded as copies/μl. The percentage of *A. racemosa* present in the engineered sample mix was calculated by normalizing the 100% *A. racemosa* gene copy number with the external /exogenous calibrator gene copy number. This ensures the advantage of using the exogenous calibrator by limiting the inhibitory effects of the primers and probe of the *A. racemosa* assay. The data normalization process was carried out in two stages. First, the copy number of the control sample obtained using the exogenous calibrator gene was normalized by itself to 1. Next, the test samples were normalized by the control sample of the exogenous calibrator gene using the formulae (1, 2).

N= CE (control) **/** CE (control) (1)

Where N is the normalized value of the control sample, CE (control) is the copy number of the control sample (exogenous calibrator spiked 100% *A. racemosa* amplified using exogenous calibrator probe) amplified using exogenous calibrator gene.

N*_n_* = CE (Test*_n_*) **/** CE (control) (2)

Where *Nn* is the normalized value obtained for each of the test samples, calculated by substituting the copy numbers of an individual test sample with the control sample obtained from experiments using the exogenous calibrator.

The *N* and *Nn* values obtained from formulae 1 and 2 are used further for the normalization of the copy numbers of control and test samples obtained using *A. racemosa* gene.

K= CT (control) **/** N (3)

Where *K* is the normalized copy number of the control sample calculated by substituting the copy number of the control sample [CT (control)] obtained by using the target gene and the normalized value of the control sample obtained using the exogenous calibrator (formula 3).

K*_n_* = CT (test*_n_*) **/** N*_n_* (4)

Where *Kn* is the normalized copy number of test sample ‘*n*’ calculated by substituting the copy number of the test sample ‘*n*’ (from various mix percentages) [CT (test*n*)] amplified using target gene, and *Nn* is the normalized value of the test sample ‘*n*’ obtained using the exogenous calibrator (formula 4). The actual percentage of the target DNA present in a mix is the ratio of test/control samples calculated by using equation 5.

Percentage = (*Kn*/*K*) *100 (5)

**Reference List**

1. Newmaster, S. G., Shanmughanandhan, D., Kesanakurti, P., Shehata, H., Faller, A., Noce,D., Lee, J.Y., Rudzinski, P., Lu, Z., Zhang, Y., Hanner, R., Ragupathy, S., & Swanson, G. Recommendations for validation of real-time PCR methods for molecular diagnostic identification of botanicals. *J. AOAC Int*. DOI: 10.5740/jaoacint.18-0321 (2019).
2. Bustin, S. A., Benes, V., Garson, J. A., Hellemans, J., Huggett, J., Kubista, M., Mueller, R., Nolan, T., Pfaffl, M.W., Shiply, G.L., & Vandesompele, J. The MIQE guidelines: minimum information for publication of quantitative real-time PCR experiments. *Clinical Chem. DOI:* <https://doi.org/10.1373/clinchem.2008.112797> **55,** 611-622 (2009).
3. Pfaffl, M. W. A new mathematical model for relative quantification in real-time RT– PCR. *Nucleic Acids Res.* **29,** e45-e45. DOI: <https://doi.org/10.1093/nar/29.9.e45> (2001).
